# Prophylaxis and management of postoperative nausea and vomiting in enhanced recovery protocols: Expert Opinion statement from the American Society for Enhanced Recovery (ASER)

**DOI:** 10.1186/s13741-016-0029-0

**Published:** 2016-03-02

**Authors:** Ruchir Gupta, Roy Soto

**Affiliations:** Stony Brook University School of Medicine, Stony Brook, NY USA; Oakland University William Beaumont School of Medicine, Royal Oak, MI USA

**Keywords:** Nausea, Vomiting, Prophylaxis, Expert Opinion

## Abstract

International experience and evidence-based practices have shown that reduction in variability through use of protocolized perioperative care improves surgical outcomes and reduces costs to patients and healthcare systems. In this series of Expert Opinions, we provide consensus recommendations for the various components of perioperative care to aid with the development of enhanced recovery after surgery protocols.

## Main text

### Parameter definition

Early ambulation, early feeding, and early return of bowel function are linked with quicker recovery, earlier discharge, and improved satisfaction. Postoperative nausea and vomiting (PONV) is a common complication following abdominal surgical procedures with patient (young age, female sex, history of motion sickness), anesthetic (use of opioids and volatile anesthetics), and surgical (length of case, use of insufflation, manipulation of the bowel) factors contributing to its incidence and severity. Nausea and vomiting should be viewed as existing on a continuum, and sequelae and patient discomfort vary based on patient experience, surgical procedure, and recovery profile. Guidelines for its avoidance and treatment are based on evidence-based literature, expert opinion, and professional association recommendations [ASPAN’S evidence-based clinical practice opinion for the prevention and/or management of PONV/PDNV. J Perianesth Nurs 2006 [Gan et al. 2007] Gan et al. 2014. Gan et al. 2003. 

### Discussion

Prophylaxis of PONV is based on a number of risk factors [Apfel et al. 1999, presented here as modifiable and non-modifiable:

1. Modifiable risk factors
  - Use of volatile anesthetics
  - Intraoperative use of opioids
  - Surgical/anesthetic time
  - Smoking (tobacco use decreases PONV risk)
2. Non-modifiable risk factors Apfel et al. 2012a
  - Female sex
  - Young age (<50)
  - History of PONV
  - Surgical location (abdominal/pelvic, ENT, breast)
  - Surgical technique (laparoscopic)

The number of medications/techniques used for prophylaxis is dictated by the numbers of risk factors present. Patients with ≤1 risk should be considered at low risk, and those with ≥3 should be considered at high risk [Apfel et al. 2012b]. Prophylaxis and treatment should rely on modification of anesthetic technique, if possible, and the use of medications that work at a variety of receptor sites allowing for full multimodal benefit. Non-pharmacologic techniques include ensuring appropriate analgesia and hydration, acupuncture/acupressure, and aromatherapy. Pharmacologic techniques include medications that work at different receptor sites:

1. Serotonin antagonists (e.g., ondansetron)
2. Dopamine antagonists (e.g., droperidol, metoclopramide)
3. Anticholinergics (e.g., scopolamine)
4. Antihistamines (e.g., diphenhydramine)
5. Steroids (e.g., dexamethasone)
6. Others (e.g., trimethobenzamide)

## Conclusion

### ASER Expert Opinion

All patients should receive PONV prophylaxis during the perioperative period. The numbers of medications used for treatment and prophylaxis should be determined by the number of modifiable and non-modifiable risk factors. Medications used should represent different mechanisms of action in an attempt to achieve multimodal benefit Tramèr 2001.

## Abbreviations

ASER: American Society for Enhanced Recovery
ENT: ear, nose, and throat
PONV: postoperative nausea and vomiting
