# Erratum to: ‘Prophylaxis and management of postoperative nausea and vomiting in enhanced recovery protocols’

**DOI:** 10.1186/s13741-016-0034-3

**Published:** 2016-05-03

**Authors:** Ruchir Gupta, Roy Soto

**Affiliations:** Stony Brook University School of Medicine, Stony Brook, NY USA; Oakland University William Beaumont School of Medicine, Royal Oak, MI USA

## Erratum

Unfortunately, the original version of this article (Gupta & Soto 2016) contained an error. The title was stated incorrectly. The original title: “Prophylaxis and management of postoperative nausea and vomiting in enhanced recovery protocols: Expert Opinion statement from the American Society for Enhanced Recovery (ASER) ” should be “Prophylaxis and management of postoperative nausea and vomiting in enhanced recovery protocols”
